# Experiences of transition from hospital to community living via the Pathways to Community Living Initiative: A qualitative evaluation study of service users and family members

**DOI:** 10.1177/10398562231211126

**Published:** 2023-11-02

**Authors:** Peri O’Shea, Kathryn E Williams

**Affiliations:** Australian Health Services Research Institute, 8691 University of Wollongong, Wollongong, NSW, Australia; Australian Health Services Research Institute, 8691 University of Wollongong, Wollongong, NSW, Australia

**Keywords:** severe mental illness, institutionalisation, recovery, rehabilitation, patient experiences

## Abstract

**Objective:**

People with severe and persistent mental illness (SPMI) may be excluded from community-based care if their complex support needs cannot be met and are at risk of institutionalisation. The Pathways to Community Living Initiative (PCLI) aims to address barriers to community living. This study evaluated the PCLI from the perspective of service users and family members.

**Methods:**

Evaluation questions were explored in semi-structured interviews. Transcripts were coded inductively and deductively. This article adheres to relevant EQUATOR standards for qualitative research and reporting.

**Results:**

There were 37 interviews with 27 service users and 12 family members. Factors associated with positive experiences of transition from hospital included detailed planning, personalised care, and staged transitions which alleviated concerns around safety, support, and coping. Community living provided opportunities to exercise greater choice and control in everyday life and, for some, to reconnect with family. Poor physical health and social isolation were noted as potential risks.

**Conclusions:**

Participants regarded community living as preferable to hospital settings, and highly valued their freedom. They reported that clinical, aged care and disability supports helped them. Additional support may be required to improve physical health and social connectedness, and families appear to have unmet needs for psychosocial support.

Since the 1980s, there has been significant reform towards deinstitutionalisation^1^ and studies have demonstrated improvements in quality of life once people transition to the community.^2–4^ Nevertheless, many people with severe and persistent mental illness (SPMI) and complex needs remain at risk of spending long periods in hospital.^5,6^ Long hospital stays lead to institutionalisation – a loss of independence, autonomy, control, and skills.^7^ Service users often find it difficult to live in the community due to lack of appropriate care and assistance. The combination of institutionalisation and poor community-based supports compounds the difficulties of transition out of hospital, contributing to poor outcomes which reinforce long-held views in the health sector and wider community that some people are better off ‘cared for’ in contained settings.^7^

The Pathways to Community Living Initiative (PCLI) is a coordinated state-wide mental health reform program led by the Ministry of Health in collaboration with NSW Local Health Districts (LHDs). When the program began, in 2015, it was estimated that some 380 people had been in long-stay units within NSW mental health facilities for 1 year or longer.^8^ The program aimed to facilitate transitions to community for this cohort and to prevent further long stays by reforming practice towards a contemporary, recovery-oriented model. The PCLI was independently evaluated between 2017 and 2021. Findings on transitions to community and practice change in mental health services are reported elsewhere.^9,10^ This qualitative study reports on the effectiveness of the PCLI from the perspective of service users and family members.

## Method

### Design

**Figure 1.**
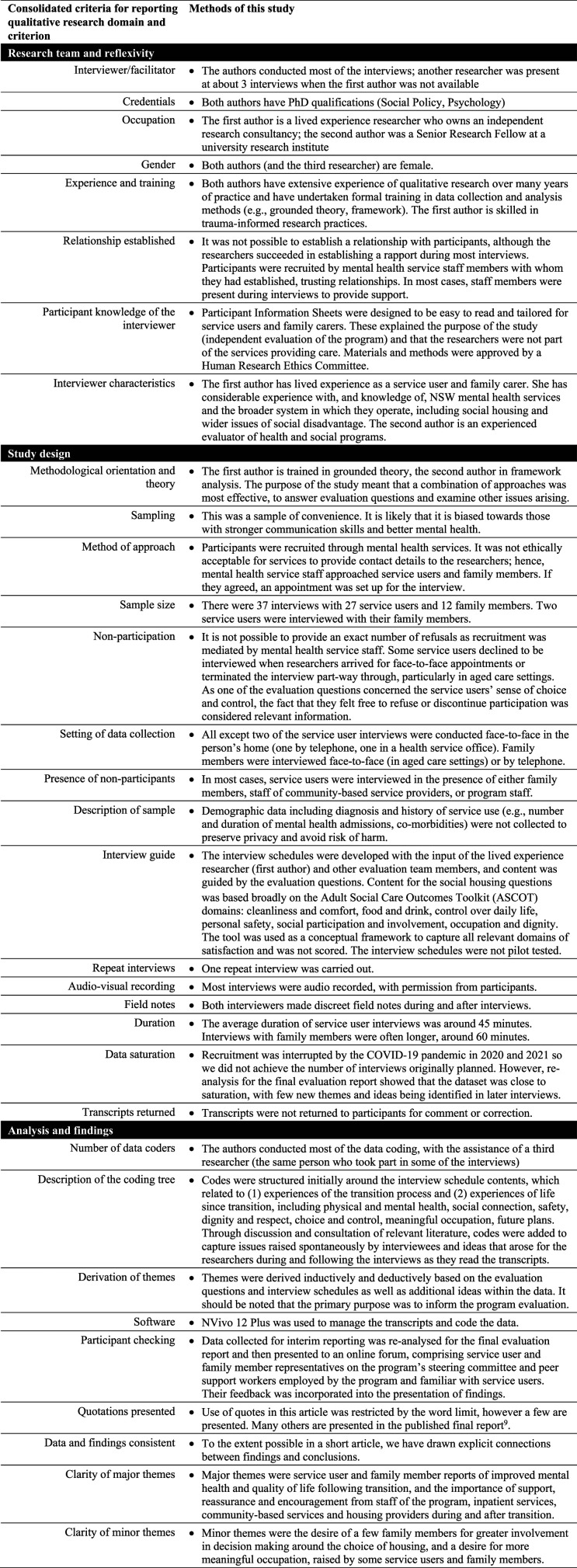
Detailed methods of this study: adherence to Consolidated criteria for reporting qualitative research guidelines.

Consistent with best practice^11,12^ and the principles of mental health recovery,^13^ and to increase the relevance and validity of results,^14–16^ this study was conducted with a lived experience academic. The study received ethics approval in May 2018. This article adheres to the COREQ^17^ standards for qualitative research (Figure 1).

### Materials

Interview schedules included experiences of transition processes; satisfaction with housing; changes in social participation, choice and control, health and wellbeing; contact with family and friends; and engagement with services. Some content was based on the Adult Social Care Outcomes Toolkit (ASCOT^18^) and a communication aid (Talking Mats^19^) was used. Diagnosis, service use history, and demography were not collected to preserve privacy.

### Procedure

Most interviews were conducted by the lived experience academic and the project manager who was accredited to tailor and use the communication aid, within the first year following transition to facilitate accurate recall. Settings for service user interviews were chosen by participants, usually their homes. Some family members were interviewed by telephone.

### Participants

Inclusion criteria were service users who had transitioned from hospital to the community or family members of service users who had transitioned. Participants were recruited via program staff in participating LHDs, with Easy-Read information sheets. This was done multiple times, starting in mid-2018. Informed consent procedures were executed at initial contact, when the appointment was made, at the start and throughout the interview. COVID-19 limited recruitment and data collection in 2020 and 2021.

### Data preparation and analysis

Interviews were recorded (with permission) and transcribed. Coding and analysis were conducted by a team with experience of the PCLI program, using NVivo 12 Plus. Inductive and deductive approaches were used to capture relevant evaluative information while allowing for the emergence of ideas and insights from interviews.

## Results

### Sample

There were 37 interviews with 27 service users and 12 family members between July 2018 and May 2021. Two service users were interviewed with their family members.

### Experiences of transition

Most service users had a clear recall of events and feelings around the time of transition. When transition was proposed, many were excited, but some worried about how they would cope and whether they would be safe. Family members tended to be anxious about the service user’s safety and access to support. PCLI teams and other hospital staff helped address concerns by providing information, reassurance, and time to adjust, while peer workers played important roles in encouraging people to think about the future and feel more positive.

Visits to proposed housing were the main source of information to aid decision making along with photos and videos. Some family members also visited proposed housing. A few family members would have preferred more information and to have been more involved in decisions around the choice of housing. However, almost all acknowledged the detailed planning and personalised care provided.

Most service users were transitioned in a staged manner, which they preferred. Gradual transitions, with opportunities for visits and overnight stays, helped people to adapt and feel confident, and were reassuring for families.

### Living in the community

Service users with issues of ageing moved into aged care facilities, others into group homes with 24/7 support, and a few transitioned into public housing with regular, drop-in support. After transition, service users liked having a private, comfortable space to themselves. Although the care in hospital was appreciated, housing in the community was universally regarded as far preferable to hospital settings.

Transition into the community provided opportunities for some to reconnect with family, including children, as they could visit in more pleasant and ‘normal’ environments compared with hospital facilities.

Most service users spoke about an increase in choice and control, which they described as ‘freedom’. Even people who transitioned into institutional environments, such as aged care, spoke about an increase in freedom compared to hospital. For example, older people said they enjoyed being able to go to their room and lie down during the day, which had not been permitted in some hospitals.

*Best thing I ever done. I’m happy. … I like to run my own life before I die. (Participant living in aged care)*

*Not saying the hospital was bad, but it’s that – there’s, you know, yeah, the one-on-one contact that families and close friends have, it’s – it just makes it a really homey feel. (Family member)*

Most service users reported that the staff in aged care or group homes treated them with dignity, respect, and kindness. They valued the conversations they had with staff and other residents. Some spoke about the challenges of learning to take personal responsibility and said that they received encouragement from staff, without feeling harassed or ordered about.

*I need a bit of a nudge every now and again … yeah, because I sometimes get a bit lethargic. (Participant living in group home)*

Some reported a decline in physical health – most commonly, weight gain – and this was often attributed to poor choices including increased consumption of junk food or smoking. Several people said they were working towards habit changes.

*I buy vegetables but I just don’t get motivated to chop it all up. … I want to start cooking more stir fries. (Participant living in group home)*

Service users were less likely to feel ‘at home’ in the community if they were socially isolated, for example, living alone or in locations with poor public transport links. Several hoped to eventually to live more independently or return to a previous home.

*Some of them want to stay here forever. But I don’t want to stay forever, to be honest. (Participant living in a group home)*

Family members reported a sense of relief and improvements in their own health and well-being following transition, including less anxiety about the service user and more time for their own needs. Most preferred regular communication with community providers and the opportunity to take part in decision making, but some found involvement difficult due to their own health issues, past experiences of trauma, psychological distress, or grief. Transition resulted in some new stressors, including aged care costs and guardianship issues. Most family members did not appear to have robust support mechanisms in place. Support available from the community-based providers was mostly informal and usually directly related to meeting the service user’s needs.

## Discussion

Overall, participants in this study reported positive experiences of transition to community living with the support of PCLI staff and processes. They were grateful for a more satisfying life and greater freedom while retaining valued clinical care and functional supports. Consistent with other studies,^2–4^ service users in this study reported improved quality of life in the community. This included opportunities to exercise greater choice and control over their daily lives, reconnect with family and make new social connections.

With psychosocial support through National Disability Insurance Scheme (NDIS), consumers were going out in their communities to engage in regular activities. Some consumers were undertaking training, volunteering or paid work. However, the level of opportunities for participation varied depending on the type of support and resources provided within different housing settings and the age of the person. A greater focus on active rehabilitation and meaningful occupation following transition may be beneficial, both in general and particularly for older consumers who are not eligible for NDIS community access funding. Aged care providers noted that older consumers were generally more mobile and less physically dependent than the average aged care resident and therefore in greater need of activities and social engagement. Programs or resources to offer activities that would promote personal recovery might assist in building and sustaining community living.

Most service users reported that their mental health and well-being had improved since transition. Some reported declined physical health, either due to ageing or to lifestyle changes. Support was available (e.g. from the staff of Supported Independent Living (SIL) providers) but there was an evident tension between encouraging healthy behaviours while supporting independence. Given the high prevalence of chronic illness and early mortality among people with SPMI,^20^ strategies to help community-based providers to support consumers to make healthier choices are needed.

Although many were initially anxious, family members felt relieved following transitions and their own health improved. New expectations of family involvement in some community settings – especially aged care – were difficult for some to sustain due to their own ageing and health issues. More support for family members would be helpful at transition and post transition stages to ensure they can continue to support the service users once in the community. In addition, further strategies and guidelines might be useful to ensure participation of service users and family members in decision making around transition.

Participants were recruited via PCLI staff teams to avoid breaching ethical requirements around the privacy of service users’ contact details. It is likely that the sample was skewed towards people with better communication skills and greater willingness to interact with researchers. Nevertheless, this study has enabled a significant group of vulnerable service users to have a say about their experiences of care. While there is more to learn and much to do, this study demonstrates that with the appropriate support and housing, people with SPMI can live and thrive in the community.
